# MedSMA℞T Adventures in PharmaCity Game: Youth Experiences and Recommendation for Use in Opioid Safety Education

**DOI:** 10.3390/pharmacy11050143

**Published:** 2023-09-10

**Authors:** Olufunmilola Abraham, Courtney R. Koeberl, Tyler J. McCarthy

**Affiliations:** Social and Administrative Sciences Division, Madison School of Pharmacy, University of Wisconsin, Madison, WI 53705, USA

**Keywords:** opioid, adolescent, serious game, medication responsibility, medication safety

## Abstract

Adolescents are often excluded from the creation of opioid safety interventions; therefore, it is crucial to design evidence-based interventions tailored for and with youth. Video games are ubiquitous and approachable to adolescents making them an accessible educational modality. MedSMA℞T: Adventures in PharmaCity is a serious game that educates adolescents and their families on the safe, appropriate, and responsible use of opioid prescriptions. The first objective of the study was to elucidate adolescents’ experiences and perceptions of the game. The second objective was to elicit their recommendations for use and suggestions for improvement. Adolescents were recruited through Qualtrics research panels, social media, listservs, and snowball sampling. Recruitment occurred between April 2021 and October 2021. Eligible adolescents played the game and completed a follow-up virtual semi-structured interview with a study team member. Interviews were transcribed verbatim and uploaded to NVivo for data analysis. A thematic content analysis was performed. A total of seventy-two adolescents participated. Analysis yielded four themes: prior gaming experience, educational salience, game design impressions, and recommendations for improvement. Most adolescents approached MedSMA℞T with prior gaming experience. The youth correctly identified the game’s intended objective: the promotion of opioid medication safety. Adolescents had overarchingly positive impressions of the game’s levels, characters, and graphics. Study participants suggested expanded game levels, improved controls, and more instructions for gameplay. In summary, adolescents had favorable experiences using the MedSMA℞T game which allude to the wide-spread acceptability of this intervention among young people.

## 1. Introduction

The opioid epidemic continues to challenge United States healthcare systems and public health divisions. The current crisis is a multi-faceted, complex phenomenon which will require diagnostic, intervention, and prevention development across disciplines [1]. While opioid prescriptions are necessary in some cases, each prescription generates a risk of misuse [2]. This epidemic continues to grow and evolve, despite changing prescribing practices and a contemporary focus on heroin and fentanyl [3,4]. In 2018, death rates in the United States related to car crashes, cancer, or cardiac disease were all lower than the rate of death from drug overdose [5]. The magnitude of the opioid epidemic led to over 50,000 deaths in 2019 [6]. The subsequent COVID-19 pandemic exacerbated the already accelerating crisis [7]. For 2021, the toll of opioid misuse and overdose reached 80,816 deaths [8]. The wide-reaching effects of this epidemic leave few groups without significant impact. In 2016, 8.7% of adolescents in the twelfth grade had misused opioids at least once [9]. The median number of monthly overdose deaths for adolescents aged 10 to 19 years old increased by 109% from 2019 to 2021. Among those aged 14 to 18, the increase in overdose death was 94% from 2019 to 2021 with an additional increase of 20% from 2020 to 2021 [10].

Often, when adults are prescribed opioids for appropriate reasons, they are unequipped with the knowledge of how to safely store opioids and dispose of unused medication [11,12]. A lack of knowledge of this magnitude is particularly worrisome when the people who are prescribed opioids are parents or caregivers of youth. Adolescents have been shown to be at increased risk of misuse, opioid use disorder, and overdose compared to adults [13,14]. Furthermore, the young individuals that use prescribed or illicit opioids are at increased risk for reduced academic performance and lifelong challenges including polysubstance use disorders and unemployment [15,16,17]. Adolescents who misuse opioids are more likely to participate in other risky behaviors such as unsafe sex, physical violence, and suicide attempts [17]. In 2019, 3% of adolescents aged 12 to 17 years old in the United States reported having misused opioids in the past year [16].

Adolescents often believe that prescribed opioids are safer than common illicit opioids such as heroin and fentanyl [18]. The Partnership for a Drug-Free America conducted a 20 million participant survey during 2008 and found that 41% of adolescents believed that prescription medications are safer than illegal ones [19]. In the same study, of the 20 million adolescents who completed the survey, 29% believed that prescription medications used for analgesics were not addictive. Over 50% of adolescents who completed the Drug-Free America survey stated that it was easier to obtain these drugs from a medicine cabinet in their family’s house than to receive illegal drugs from dealers [19]. Parents who are unaware of safe storage and disposal practice model unsafe behaviors in the home [20,21]. Coupled with youths’ misunderstandings about opioids, it is critical to design novel interventions that are tailored to and target adolescents. Furthermore, it is critical to target adolescents through media that are acceptable, usable, enjoyable, and attractive to them.

Video games have the potential to be more than a source of entertainment [22]. One genre of video games includes serious games, and these can improve cognitive and psychosocial skills by educating their players on new topics [23]. Specifically, serious games are games whose primary objective is not mere entertainment [24,25]. Considering that nine out of ten American youth (children and teens) play video games, it is worthwhile to design, test, and refine serious games in familiar formats [26]. Serious games have demonstrated acceptability amongst youth for health promotion [27,28]. Some studies indicate the efficacy of serious games for the promotion of healthy behavior and knowledge for youth with chronic conditions and youth generally [29,30,31]. Other games have been created to target opioid abuse or vaping prevention, for example [32,33].

MedSMA℞T: Adventures in PharmaCity (MedSMA℞T) is a serious game designed to teach adolescents about core prescription opioid safety principles and encourage conversations around medication safety in the home. While some serious games have covered the topic of opioid safety, MedSMA℞T is the first to leverage a robust scientific approach to co-design a family-based intervention for dissemination in pharmacies and clinical settings. The game takes players through a responsive narrative wherein they make decisions while playing as the main character. Shan, the sheep, is the game’s protagonist. They are a teenage, anthropomorphized sheep who has recently broken their arm; it is up to the players to help Shan manage their opioid prescription safely and appropriately throughout the week. Level 1 confronts players with a situation where Shan’s friends spot a prescription left on the kitchen counter. It is up to players to safely store the opioids to keep them out of reach of others. Level 2 is a scene on the bus where Shan learns that they have forgotten about an important assignment. Level 3 takes place at school and Shan must make safe decisions to earn a good grade on their speech and sit with their friends at the big game. Level 4 is another bus ride back home where a rider is in pain and asks Shan if they have medication to spare. In this level, players are taught to keep their medicine for themselves. In the fifth and final level, players must help Shan dispose of their opioid prescription in the safest way possible. Players are confronted with a range of disposal options; however, to complete the game, they must take the prescription to the pharmacy’s drop box.

This serious game was designed following studies conducted by the research team with adolescents, parents, pharmacists, game designers, addiction medicine specialists, and adolescent health experts. Studies elucidated adolescents’ preferences for education as well as practical gaps related to safe prescription opioid behavior and knowledge [34]. A psychometrically validated questionnaire (AOSL) was developed to assess youths’ knowledge of opioid prescription safety practices and general opioid knowledge [35]. After engaging with adolescents and young adults (AYAs), the study team utilized their perspectives to create MedSMA℞T: Adventures in PharmaCity. Initial playtests engaged AYAs to refine the game’s prototype, demonstrating the salience of game objectives and early acceptance of this intervention in terms of design features and realism [36,37]. Further evaluation with a national sample of youth demonstrated that MedSMA℞T can improve adolescents’ opioid knowledge, prescription disposal knowledge, behavioral intent, and self-efficacy related to opioid safety behaviors [38]. The study team also engaged with pharmacists, eliciting their feedback on the game, its contents, and implementation factors. Pharmacists reported positive recommendations of the MedSMA℞T game, noting the interactive gameplay, educational value, and appropriateness for the target audience. They believed that the game would be feasible to be implemented in the community pharmacy setting and would provide a more robust consultation than what they could provide at the counter [39]. Thus, it was important for the study team to obtain adolescents’ perceptions of using the developed game. 

The primary objective of this study was to characterize adolescents’ perspectives on MedSMA℞T: Adventures in PharmaCity to understand their experience with gameplay and medication management. The second objective was to elicit suggestions to inform refinement toward adoption, acceptability, and quality.

## 2. Materials and Methods

This study was approved by the Institutional Review Board (IRB) at the University of Wisconsin–Madison before study recruitment and data collection began.

### 2.1. Participants and Recruitment

Recruitment occurred through Qualtrics research panels, snowball sampling, social media, and email listservs. The study team worked with Qualtrics to recruit a national sample of youth. Qualtrics maintains preexisting research panels who were contacted via email for study participation. Email lists came from the university’s mass email system and the study lab’s own listserv. Eligible adolescents were those between the ages of 12 and 18, who lived in the United States, and could read, speak, and understand English. Since the study took place virtually, through WebEx, it was also required that the youth had access to the Internet and a computer with a webcam. Interested participants filled out a Qualtrics interest survey and were contacted for up to three weeks via email afterward by a study team member to schedule a study session. Participants were encouraged to schedule the study session at a time and in a location where they felt the most comfortable. Participants could be in any location of preference while in the study session. Adolescents and their parents were recruited for the study since parental informed consent was required and obtained prior to adolescent assent. Parents were given a USD 30 Amazon e-gift card for their child’s participation.

### 2.2. Data Collection

Data were collected virtually through WebEx and participants were audio–video recorded. Adolescents were asked to complete the study in a room away from their parents to allow the youth to generate their own perceptions of the game. At the start of the study session, eligibility and consent (or assent) were once again confirmed. Then, participants were instructed to play 30 min of MedSMA℞T while a study team member observed for troubleshooting assistance or data collection errors. The study team member would not intervene unless there was an issue causing the game to become unplayable. This did not occur during any study sessions. Additionally, the study team member turned off their camera and audio during the gameplay stage to further limit bias that could come from interviewer vocalizations or facial expressions. 

Afterward, participants completed a 30 min semi-structured interview about their experience throughout the game. The interview guide covered the design characteristics of the game and its content, what the participant perceived the purpose to be, and the adolescents’ experiences of different games. The interview guide is reported in Appendix A. Both the gameplay and interview sessions from the participants were recorded for data verification and audit trail creation. Interviews were transcribed verbatim by a professional transcription company. Interviews were checked by a research team member for accuracy and all potential identifiers were removed prior to analysis.

### 2.3. Data Analysis

Study team member CK performed the data analysis. The data were analyzed using NVivo (QSR) software. Data were investigated using a thematic content approach to reveal manifest codes related to adolescents’ experiences with gaming and the MedSMA℞T game [40,41]. The study team member began by familiarizing herself with all of the transcripts by closely reading each transcript. Following this, CK developed preliminary codes. Next, a preliminary code book was developed and checked by OA. Then, CK completed the coding of all of the transcripts and exported the data to an Excel spreadsheet to extract code counts by file and number of references. Themes were developed by sorting codes based on their occurrence across transcripts. A data audit was conducted by a study team member (TM) not involved in preliminary analysis to ensure that all transcripts were analyzed. Prevalent codes were identified using NVivo software and then aggregated into major themes and subthemes supported through verbatim quotes from the interviews. 

## 3. Results

A total of 72 adolescents completed both the gameplay and interview component of the study. Participant characteristics are reported in Table 1. Notably, participants were more frequently male, white, and monolingual English speakers. Following data analysis according to the study theme, four main themes described below emerged, which include prior gaming experience, learning outcomes, game design impressions, and recommendations for improvements. 

### 3.1. Prior Gaming Experience

#### 3.1.1. Preferences for Gaming

Adolescents were asked about their prior gaming experiences and preferences. Most participants indicated that they had at least some experience playing games. Most participants recounted their experience with video games and fewer participants described playing board or card games in the past. Frequently, adolescents articulated a preference for games that allow for creativity, building, exploration, and competition. Adolescents most frequently reported playing games on computers or consoles with fewer reporting playing mobile games or board games. A smaller number of youths suggested that they do not regularly play video or board games due to a lack of interest or time.

“As for Minecraft, the same thing, I like crafting things. I like strategy. I like survival. I like being able to, you know, explore new things.”—Adolescent 1, age 14.

“No, I just don’t like enjoy staring at a screen for very long.”—Adolescent 2, age 16.

#### 3.1.2. Reasons for Gaming

Adolescents offered a variety of reasons as to why they choose to play certain games. The most frequent reasons were enjoyment, challenging oneself, or socializing with friends or family. Playing with friends was the most frequent reason that adolescents gave for playing games. Some of the youth talked about how games provided a means of socialization during the COVID-19 pandemic. Less frequently, adolescents mentioned playing games because of the perceived autonomy given by virtual spaces, because they wanted to improve their cognitive skills or strategic abilities, and because of the ease of gameplay. 

“So that I can talk to my friends online, especially during this last year of COVID.”—Adolescent 3, age 13.

“I have experience playing video games. I find them as enjoyable things that you can do with other people and a fun way to pass time.”—Adolescent 4, age 12.

“Currently I play some shooting games because I feel like it’s, it helps me with like my reflexes kind of. I know that’s kind of strange, but it kind of helps me with like my reflexes and my awareness, you know peripherals and stuff like that.”—Adolescent 5, age 15.

“Um, I don’t play games on my phone, but I do play board games, like chess and checkers sometimes because, (1) they’re fun (2) uh, they’re good for training…your cognitive abilities.”—Adolescent 6, age 13.

“I do play board games, some card games, just for the interaction with like other people and the change to strategize to eventually win the game.”—Adolescent 7, age 15.

#### 3.1.3. Perceptions of Educational Games

Participants were asked to describe their experiences using educational games as well as name those that they had played in the past. Very few participants were able to recall the name of the educational games that they had played in the past. Some participants recounted specific topics such as language, politics, mathematics, or science. Divergence in adolescents’ impressions of educational games was suggested to be due to a game’s enjoyability, topic salience, and location of gameplay. Some students suggested that they had positive experiences with educational games and would choose to play them. Many adolescents articulated that educational games can be enjoyable and a quality resource for new learning. A few participants suggested that educational games were boring or difficult for them and were only likely to play games assigned in school. Disinterest and difficulty were often expressed when adolescents described the game as being poorly designed or information-dense. A small number of participants indicated no prior experience with educational games. However, regardless of prior experience, adolescents suggested they were willing to play educational games. 

“I feel like educational games that we play in school are usually, like they try to be full normal games, but then they just implement too much, I guess, of the main idea really, and it just makes it really unfun, and it just kind of makes it feel like it’s just information with a side of game.”—Adolescent 8, age 12.

“So I like playing educational games because it shows that, you know, education may be boring, and even though there’s good things to it, there is a way to make education fun for people instead of sitting behind a whiteboard and just taking whatever your teacher says for two hours.”—Adolescent 1, age 14.

“I’m, I love it as long as they’re fun to play. I think it really helps the experience because you can feel like you’re doing something productive and not just playing a game.”—Adolescent 9, age 15.

“I’ve only played, one, in the past. And it wasn’t very entertaining. It was just basic, bare bones didn’t really have anything added on to it.”—Adolescent 10, age 14.

### 3.2. Educational Salience

#### 3.2.1. Perceived Purpose

Most adolescents perceived the game’s purpose to be centered around the safe usage of opioids. Many participants stated more specific purposes such as proper storage or disposal and not sharing medication. Other youths conveyed that the purpose to them was showing consequences of misuse, encouraging responsibility with medications, or to keep others safe from opioid misuse. 

“The goal of the game is to inform people on what, on what can happen in you (A) don’t secure your meds (B) take meds that aren’t yours. Uh. And (C) don’t dispose of the meds properly.”—Adolescent 6, age 13.

“I think the goal is to how to live your life safely when different situations come up.”—Adolescent 11, age 14.

“I felt like the main goal of the game was to generally explain what to do in situations that will probably, more than likely, happen in life sometimes, so you can be aware of that and what to do in those situations.”—Adolescent 12, age 13.

“I thought the main purpose was to tell people how to use, or like, like someone who’s taking opioids, like how to use them, how to dispose of them, what they should do if they have them just to be responsible and stuff.”—Adolescent 13, age 14.

“To teach people about the steps to take when you’re prescribed a painkiller, medication, and like, how to be safe with it. And what to do when you’re done with it.”—Adolescent 14, age 13.

#### 3.2.2. Learning Outcomes

Adolescents were asked what they had personally learned from playing MedSMA℞T. Nearly all of the participants indicated that they had learned something new. Some adolescents reported that the information that they gained from playing the game was similar to their stated perceived purpose of the game. Most adolescents offered specific takeaways with the most frequent response being proper storage or disposal. Many participants suggested that they were unaware of medication drop boxes and that unused or expired opioids could be disposed of in them. Another common lesson learned was not to share opioids with others under any circumstances. Some adolescents suggested that they learned what opioids were and the dangers of misuse. Very few adolescents reported that they did not gain any new information from playing the game, and those who did suggested that they had prior knowledge on the topic. Of those who did not report learning something new, all except for one adolescent were able to accurately identify the game’s purpose. 

“I learned that you shouldn’t take opioids from other people. I didn’t even know like opioids were a thing before playing that.”—Adolescent 15, age 14.

“I liked the message. And I think that before I played the game, I really didn’t know how to dispose of opioids or pain medicine. And after the game, I was educated on, you know, putting them in a drop box or returning them to the pharmacy if they’re expired.”—Adolescent 11, age 14.

“I guess I learned about the disposal boxes. I didn’t know too much about those being a thing. So it’s probably not too much of a good idea to just toss them in the trash or willy-nilly.”—Adolescent 16, age 15.

“I didn’t really learn anything that I didn’t already know.”—Adolescent 17, age 14.

### 3.3. Game Design Impressions

#### 3.3.1. Characters

Taking a closer look at the characters of MedSMA℞T, most participants agreed that they were realistic. Few participants thought that the characters were not realistic, but the game still presented an informational storyline. Overall, the characters were described as likable, good, cute, and funny. Many enjoyed the use of animals as characters. 

“Oh, I liked the characters. You know, they learned as, like with the game. It’s not like they knew everything. They, some made mistakes…They seemed pretty realistic.”—Adolescent 30, age 16.

“I liked the characters. I also like the fact that it wasn’t necessarily like female or male. Like I didn’t focus on that or anything.”—Adolescent 11, age 14.

“They [the characters] seem relatable, but a few of them seem static. They don’t really seem to develop, and they just seem to be carbon copies of normal tropes. But in a game like this, that doesn’t really matter. It’s not really based around story. It’s based around opioids and their consequences, and they should not be abused in any way.”—Adolescent 18, age 16.

#### 3.3.2. Graphics

The majority of adolescents had favorable feedback regarding the game’s graphic design. The graphic design was liked by many for its 2D artistic style. A few participants mentioned that they were less interested in the artistic style, describing it as lower quality and more similar to older styles when compared to the graphics of contemporary, popular games.

“Ah, I thought some of the humor and communication through some of the people was ah, accurate and humorous. I like the art style, it’s kind of cute and cartoony.”—Adolescent 19, age 17.

“I liked the graphics. I thought it was pretty cool art like. I liked how it was 2D.”—Adolescent 20, age 14.

#### 3.3.3. Gameplay

Participants reported positive experiences with the MedSMA℞T gameplay. Adolescents articulated that the responsive narrative format (having multiple choices in the storyline) was engaging and enjoyable. Many adolescents suggested that the storyline and levels within the game were realistic and relatable to teens. 

“What I liked was being able to go through the scenarios based on your own actions instead of being led on how do it because when you are being led to a certain thing, you aren’t really showing what, you know.”—Adolescent 21, age 15.

“It takes a very, very relatable situation, a sports injury, and it’s able to show, for instance, what could happen if you make the wrong decision… Some of the problems I had with this game are definitely the controls.”—Adolescent 18, age 16.

“I really appreciate like how it’s not easy because, like I said, most educational games are, they’re so simple and so easy that it’s like not even really a game. It’s more of a lesson. And while this one was like still educational, it was like it was actually fun to play. And like the, it felt like you were playing a video game, not a textbook.”—Adolescent 22, age 12.

#### 3.3.4. Willingness to Recommend

Adolescents were asked if they would recommend MedSMA℞T to others. Most participants indicated that they would recommend the game to others. Of those who would recommend it, some elaborated on who they would recommend the game to and why. Adolescents conveyed reasons as to why they would suggest the game, such as its educational value or ability to make learning more enjoyable. Participants saw utility in implementing the game in clinical settings for those prescribed opioids or in schools. A few participants mentioned that they would recommend it but would not say that it was a game for recreation or entertainment. Others suggested that other age groups such as younger children or older teenagers would receive their recommendation. 

“I would recommend this game because I thought it was like really fun to play and then you could also learn things along the way as well.”—Adolescent 23, age 15.

“I would recommend the game to others because it’s very informative—informative and is needed, because schools, don’t really teach you about this.”—Adolescent 21, age 15.

“Yes, I would recommend this game to other people because it definitely is educational, and it helps spread the word and spread awareness, which I think is really important, especially nowadays because I think too many people just get hurt and in trouble and, with this kind of stuff.”—Adolescent 5, age 15.

A minority of adolescents stated that they would recommend the game to a family member or friend taking opioid medication. A few participants stated that they may not recommend the game because others would not take them seriously or may discredit the recommendation, or stated that it is not relevant to people they know. One adolescent stated that the information could be learned from other sources. 

“No, because there was boring and they’re unoriginal, but like, it’s educational, but they can get the same thing from a like, a thing online, like a, I forgot what called. I’ll know what it’s called. They could get this information literally off Google or from a doctor, or common sense, common sense is probably where it’s like, even before this, even before I talk about a patient, and if someone knows like, “hey, you want some of my pills” I’d be like, “No, what the hell.””—Adolescent 25, age 12.

“No, because anybody I’d recommended to would tell me I’m stupid for telling them, so.”—Adolescent 29, age 16.

“I probably wouldn’t only because I don’t have anyone in my life who this, this game relates to. If I did have a friend or family member who was taking opioid medication I might.”—Adolescent 25, age 14.

### 3.4. Recommendations for Improvement

While there were a handful of individuals who stated that no improvements needed to be made to the game, other study participants provided helpful recommendations. Suggestions most often referenced more scenes or game expansion, improved controls, and clarification around in-game tasks.

#### 3.4.1. Additional Game Characters, Customization, and Levels

Participants suggested expanding the MedSMA℞T game. One type of expansion was to build upon the existing branching narrative by including more options and making choices that have longer-term effects in the game. Many wanted increased options and scenes to allow for more exploration and variation within the game. Other suggestions were to add specific scenes into the game such as how to spot and respond to an overdose, and how to support someone with an opioid use disorder.

“I think that maybe adding some instances where the character will have to deal with someone who is experiencing an overdose or a family member that is experiencing an overdose.”—Adolescent 26, age 16.

“I would probably say adding more characters, more levels, being able to customize characters, making your own character, being able to, like, unlock, like, items and things to help you.”—Adolescent 27, age 13.

“And one way that I think that could be improved upon would be introducing branching paths… some of them should have more lasting consequences, instead of just, you messed up, redo, you didn’t mess up.”—Adolescent 18, age 16.

#### 3.4.2. Improved Controls

Another game design suggestion included improving the controls. A few adolescents reported difficulty learning the game’s controls. The game allows for both keyboard (WASD, arrow keys) and click-based (mouse) controls. However, the formatting of the game makes click-based play more difficult. Adolescents suggested having only one type of control or explaining the controls at the start of the game. 

“I just wish that all of the interactions were the same, and you don’t have to constantly switch between different types of controls to interact with that one thing.”—Adolescent 1, age 14.

#### 3.4.3. Clarifications of Game Features

Participants recommended clarifying certain game features. A common theme among the responses of the participants was that they also wanted to see explanations behind the answer options. When adolescents correctly (safely) navigate through a level, there is no additional explanation as to why the choices made were the best. When an incorrect (less safe) choice is made, players see the consequences of that decision. The players of the game may benefit if they are able to understand and read why they got an answer wrong or right. The youth also suggested adding features that encourage easy gameplay, such as direction arrows, a progress bar, or explicit objectives. 

“The only gripe I have about the game is that, you know I wish like in the beginning, I wish there was a bit more clarity about what, you know, where to start, where to begin.”—Adolescent 28, age 18.

## 4. Discussion

Overall, adolescents reported positive feedback on using the MedSMA℞T game with only some suggestions for improvement. The youths’ perspectives of using the MedSMA℞T game underscore the potential for this intervention to meaningfully connect with adolescents in an acceptable, enjoyable, and educative manner. All adolescents had at least some experience with video games, and a smaller number had experience playing board games. The youth in this study favored games where they could create and explore according to their own desire. The youth also preferred games that tested their strategic and competitive skills. 

MedSMA℞T is formatted as a responsive narrative which allows youth control over how the game progresses. Moreover, the searching and sequencing of the game allow for a more explorative gameplay. It is important to keep in mind the preferences of youth in designing similar serious game health interventions, thus incorporating design features that they find enjoyable and educational. Adolescents play games for a myriad of reasons, including competition, cognitive training, enjoyment, and social interaction. Many cited that online multi-player games and board games are preferable ways to build cognitive skills or interact with friends and family. Following the COVID-19 pandemic, games became affinity spaces for youth to make and maintain relationships with peers. Prior to experiencing MedSMA℞T, adolescents had already developed a stigma against educational games as being boring and unentertaining. A few adolescents vaguely remembered playing educational games in school or at home but were typically unable to recall the names or specific gameplay [42]. This is despite the potential for game-based learning to foster improved cognitive processing and motivation of the players [43].

Adolescents may have negative impressions of educational games for a variety of reasons. The most common reason stated in this study was that the games were not entertaining. While the purpose of educational games, in particular serious games, lies outside of entertainment, it is still an influential factor for engagement and learning. Studies have explored the link between entertainment and education in serious games, notably, levels of entertainment have been found to affect engagement, behavioral intention, knowledge acquisition, and length of gameplay. Through evaluating study participants’ impressions, it was found that adolescents tended to enjoy MedSMA℞T when compared to educational games that they had played in the past. From these positive impressions and adolescents’ ability to accurately articulate the game’s purpose, it is inferred that this game provides balance between education and entertainment.

From the youths’ responses, it was clear that the characterizing purpose of MedSMA℞T was salient. The youths in this study reported emerging from gameplay having learned new information about opioid safety. The youth noted that they learned about proper storage and the safe disposal of opioids [34]. Many were previously unaware of medication drop boxes or the importance of locking away opioid medications. Giving adolescents this information could encourage them to speak with their parents about adopting safe opioid medication use practices in their own homes. These takeaways are critical not only for if the adolescent is taking opioids, but their new knowledge could prove helpful to caregivers and family members who may be unaware of these safe practices themselves [12,44,45]. While adolescents are the primary audience, the information in the game could remind or teach parents about key concepts such as safe storage and proper disposal. 

Adolescents’ takeaways are not the only indicator of MedSMA℞T’s potential to encourage safe behavior. Many youths suggested that they enjoyed the game and its components. Moreover, nearly all the youth affirmed that they would recommend the game to others. While many were hesitant due to the perceived social faux pas of recommending an educational game in real life, they still suggested that they would be willing to recommend it. The youth also affirmed their support for the anthropomorphic characters and the game’s artistic style. Moreover, when asked for suggestions to improve the game, the most frequent response was to expand it. The youth wanted to see more choices, longer-lasting consequences, and more levels to attempt. These responses were promising in terms of youth acceptability, highlighting that the game is educational but enjoyable enough that they wanted more. Adolescents’ favorable experiences using the MedSMA℞T game allude to the wide-spread acceptability of this intervention among youth. Further research can explore adolescents’ ability to think of situations such as problems at school or family settings where they might be more susceptible to opioid misuse. Scenarios created by adolescents can then be incorporated into future iterations of the MedSMA℞T game.

While adolescents mostly offered suggestions to expand the game, some suggested improving the technical aspects of the game. Key adaptations following this study mainly encompass controls and directions. It may be beneficial to provide a tutorial scene that walks the players through the game’s controls so that players do not have to think about which keys to use. A tutorial would also help to orient the player to the game world. Additionally, clarifying gameplay directions could be piloted to see whether giving explicit objectives would improve the current gameplay design. Future studies are needed to assess MedSMA℞T with a more diverse sample of youth and to test for outcomes related to knowledge acquisition and behavioral intention. Moreover, future studies should elucidate the long-term impacts of the intervention on opioid knowledge and safety behaviors. Studies underway are evaluating the implementation of the MedSMA℞T Families intervention in various clinical settings, including an emergency department and community pharmacies. 

### Limitations

There are several limitations to this study. The adolescents’ responses could have been affected by social desirability bias, making their responses more positive. Additionally, the sample was generally homogenous with many participants identifying as white, male, and monolingual (English). Therefore, findings may reflect the views of a particular subgroup of adolescents, not adolescents generally. The youth of different gender, race, or socio-economic status may be more likely to exhibit opioid misuse or experience precipitating situations leading toward misuse. Therefore, it is imperative that future studies recruit a more diverse sample to evaluate the need for different scenarios or educational points for adolescents from specific backgrounds. In addition, remuneration was used in this study, making it possible that the participants were more inclined to play an educational game due to a monetary incentive.

## 5. Conclusions

The growing body of knowledge in the field of educational psychology exploring the use of gamification and simulation has shed light on the value of motivation, gameplay, or roleplay in improving health education. It is critical to include adolescents in the creation and design of healthcare interventions such as serious games to ensure their acceptability and sustainability for use in actual practice. Accordingly, adolescents desire serious games that allow for creativity, building, exploration, and competition. Educational games often have a negative stigma to them, but MedSMA℞T has been shown to take a unique approach. The overall findings from the study demonstrate that an overwhelming number of adolescent participants favored using MedSMA℞T as an educational tool to prepare adolescents for real-world scenarios involving opioids and other medications. Opioid hospitalizations and mortality may be prevented by tailoring drug safety education to a platform which adolescents will enjoy. The youth reported the MedSMA℞T serious game as being acceptable, useful, enjoyable, and educational. Future research will evaluate the effectiveness of disseminating and implementing the MedSMA℞T serious game in clinical settings such as emergency departments and pharmacies.

## Figures and Tables

**Table 1:** Sample demographics (*n* = 72).

Characteristic	Mean	SD
Age (mean (Standard Deviation (SD)))	13.89	1.61
Gender	*n*	Percent
Male	41	56.9%
Female	23	31.9%
Nonbinary	3	4.2%
Transgender	2	2.8%
Identify differently *	3	4.2%
Language	*n*	Percent
English	65	90.3%
English, Spanish	5	6.9%
English, French	1	1.4%
English, German, French	1	1.4%
Race/Ethnicity	*n*	Percent
White or Caucasian	46	63.9%
Black or African American	10	13.9%
Asian and White or Caucasian	2	2.8%
Hispanic or Latinx	3	4.2%
Other, please specify:	3	4.2%
American Indian or Alaskan Native	1	1.4%
Asian	1	1.4%
More than one **	6	8.4%
Household size under 18 (mean (SD)) [*n* = 71] ***	0.93	1.00

* Denotes participants who chose to self-identify outside of provided categories. ** Denotes participants who selected more than one of the provided categories of race/ethnicity. *** Participants were asked how many children under 18, aside from themselves, live in the home.

## Data Availability

Data are unavailable as they contain protected health information.

## Appendix A. Interview Guide

Thank you again for participating in this interview. As stated in the consent form, this interview will be audio recorded and will not be linked to any of your identifiable information. Avoid saying names of people or organizations or mentioning any sensitive topics in the interview. Feel free to stop me if you have questions at any point during this interview. You may also skip any questions you are uncomfortable answering. If you begin the interview and change your mind, you may end participation at any time without penalty. Today we are going to ask you questions about your experience playing the MedSMA℞T game. Please answer these questions honestly.

We will not share your answers to these questions with your parent/guardian. We are researchers collecting data that may help improve medication safety. Answering the following questions honestly will allow us to help improve medication safety for others.

Do you agree to continue with this interview?
The first questions are about the game and elements of the game.
What did you think of the video game?
What did you like or dislike about the game, and why?
What changes could be made to improve the game?
Tell me how you feel about the characters in the game. (Prompt, if needed: Were they realistic? What did you like/dislike about them? How could we improve them?)
How did you feel about the scenarios presented in the game? (Prompt, if needed: Were they realistic? Engaging?)
Would you recommend this game to others, why/why not?
The following questions are about what you learned from the game.
What do you feel was the main goal of the game? How could the game be improved in order to meet this goal?
What did you learn from this game, if anything?
Now, I’d like to ask you some questions about your experience with games.
Do you have any experience with playing video games? Please tell me about your experience with video games.
(If they have little to no experience): Is there a reason you don’t play video games? If yes, why not?
(If they have little to no experience): Is there anything that could be changed that would make you want to play them?
(If they do play games): What type of video games do you prefer?
What games are you currently playing? Why do you enjoy playing these games?
Do you play games on your phone/board games? If so, what games? Why do you play them?
How do you feel about playing games that are educational?
Have you played any other educational games? If so, what do you like or dislike about these games?
Is there anything else you’d like to add?
Thank you so much for taking the time to complete this interview with us. I will now stop the recording.
